# Drinking behaviour and alcohol-related harm amongst older adults: analysis of existing UK datasets

**DOI:** 10.1186/1756-0500-7-741

**Published:** 2014-10-20

**Authors:** Sarah Wadd, Chris Papadopoulos

**Affiliations:** Substance Misuse and Ageing Research Team, Tilda Goldberg Centre for Social Work and Social Care, University of Bedfordshire, Park Square, Luton, Bedfordshire, LU1 3NJ UK; Institute for Health Research, University of Bedfordshire, Room 32, Putteridge Bury, Hitchin Road, Luton, Bedfordshire, LU2 8LE UK

## Abstract

**Background:**

Older adults experience age-related physiological changes that increase sensitivity and decrease tolerance to alcohol and there are a number of age-related harms such as falls, social isolation and elder abuse, which are compounded by alcohol misuse. Despite this unique vulnerability and the fact that the number of older adults is increasing, the literature on drinking behaviour and alcohol-related harm in older adults is sparse. This article describes a secondary analysis of UK data to address this knowledge gap.

**Method:**

Secondary analysis of national statistics on alcohol-related hospital admissions and alcohol-related deaths, and data on drinking behaviour from the General Lifestyle Survey. Trends were identified by calculating percentage changes between time periods. The association between drinking behaviour and selected age groups was investigated using one way analysis of variance or chi-square tests.

**Results:**

Older adults (aged 65 and over) drink less and are less likely to exceed the recommended drink limits than younger adults. However, they are more likely to be admitted to hospital for an alcohol-related condition than younger adults and the most significant increases in alcohol-related hospital admission rates in recent years have occurred in older age groups. Alcohol-related death rates are highest amongst those aged 55–74 years old. Alcohol consumption and the prevalence of exceeding the recommended drink limits has fluctuated but not significantly increased in older adults in recent decades.

**Conclusion:**

Older adults experience high and increasing levels of alcohol-related harm and as the population ages, this is likely to put increasing pressure on health and social services. Careful monitoring and age-appropriate strategies to detect and treat older adults at risk of alcohol-related harm are required.

## Background

By 2050, 22% of the world’s population and 31% of the UK’s population is projected to be aged 60 and over [1]. While media attention and public health campaigns in England focus on alcohol misuse in younger adults, in 2009/10 those aged 65 years and over accounted for 44% (461,400) of alcohol-related hospital admissions [2] yet comprised only 17% of the population [3]. Hospitalisation rates for older adults with alcohol-related diagnoses are similar to those for myocardial infarction [4] and the cost of alcohol-related inpatient hospital admissions in 2010/11 was £825.6 m for 55 to 74 year olds compared to £63.8 m for 16 to 24 year olds [5].

Even at relatively low levels of alcohol consumption, older adults can be vulnerable to harm. This is partly because the loss of lean body mass related to ageing can reduce the volume of alcohol distribution, resulting in an increased peak ethanol concentration with any given dose of alcohol [6, 7]. The activity of the enzyme alcohol dehydrogenase, which breaks down alcohol is also significantly reduced in older adults [8], potentially increasing the amount of ethanol that reaches the bloodstream. Older adults are particularly susceptible to imbalance after acute alcohol ingestion [9] making them more prone to falls [10–12]. Alcohol interacts with many medications commonly prescribed to older adults with potentially serious medical consequences [13, 14] and it is associated with a number of other consequences particularly salient to older adults such as insomnia [15, 16] and elder abuse [17].

The prevalence of older adults at risk of alcohol-related harm varies according to the population. For example studies have found that 20% of men and 7% of women aged 65 and over living in the community in England [18], 13% of men and 8% of women aged 65 and over living in the community in North America [19] and 29% of patients aged 75 and over admitted to an acute geriatric inpatient unit in France [20] were at risk drinkers .

The increasing number of older adults and their susceptibility to the negative effects of alcohol provides a compelling reason to examine drinking behaviour and alcohol-related harm in this population but data on older adults is often incomplete and sparse [21, 22]. The aim of this study was to conduct a secondary analysis of UK data to explore drinking behaviour and alcohol-related harm amongst older adults The key research questions were:

1. Are alcohol-related hospital admissions and deaths increasing in older adults?
2. Is alcohol consumption and alcohol misuse increasing in older adults?
3. How does increasing age affect the likelihood that a person will be admitted to hospital or die from an alcohol-related condition than younger adults?

## Methods

### Design

This study involved collation and scrutiny of existing UK Government data on alcohol-related hospital admissions and deaths and a reanalysis of data on drinking behaviour from a household study. The data sources, measures and methods for the analysis are described below.

### Data sources and measures

#### Alcohol-related hospital admissions

In England, alcohol-related hospital admissions are calculated using information on patients’ characteristics and diagnoses, together with best risk estimates from published literature for the proportion of cases of a particular disease or injury that are caused by alcohol (known as alcohol-attributable fractions). For some conditions, alcohol consumption causes all cases so all admissions for these conditions are included e.g. alcoholic liver disease. These are known as wholly attributable alcohol-related hospital admissions and have an attributable fraction of one. Other conditions are partly attributed to alcohol, meaning that only a fraction of these cases can be attributed to alcohol consumption e.g. cancers of the mouth, oesophagus and liver. These are known as partly attributable alcohol-related hospital admissions and have an attributable fraction of less than one and greater than zero. Together, wholly attributable and partly attributable alcohol-related hospital admissions make up all alcohol-related hospital admissions which in this paper are referred to as total alcohol-related hospital admissions.

A hospital admission is a period of patient care under one consultant within one healthcare provider. It includes all admissions to the English National Health Service (NHS) hospitals and NHS commissioned admissions in the private sector. Alcohol-related admissions are identified where an alcohol-related diagnosis is recorded by a clinician in any of the 20 (14 from 2002/03 to 2006/07 and 7 prior to 2002/03) primary and secondary diagnosis fields in a hospital record. Where there is more than one alcohol-related condition among the diagnostic codes, the condition with the largest attributable fraction is used. The method for calculating alcohol-related hospital admissions is described in detail elsewhere [23].

Data for the period 2002/03 to 2009/10 was accessed by submitting a Freedom of Information request to the NHS Information Centre in February 2012. Data for 2010/11 was extracted from the report ‘Statistics on Alcohol: England 2012’ [24]. Alcohol-related hospital admissions have been expressed as age standardised rates to allow comparisons across time periods by controlling for differences in the age structure of the population. They give the number of events that would occur in a standard population (per 100,000) if that population had the age-specific rates of a given area. The rates are standardised to the European Standard Population [25].

#### Alcohol-related deaths

Data on alcohol-related deaths in the UK is derived from the information provided when deaths are certified and registered. The definition of alcohol-related deaths only includes those causes regarded as being most directly due to alcohol consumption. Unlike alcohol-related hospital admissions, it does not include other diseases which are partly attributable to alcohol. However, it does include all deaths from chronic liver disease and cirrhosis (excluding biliary cirrhosis), even when alcohol is not specifically mentioned on the death certificate. Apart from deaths due to poisoning with alcohol (accidental, intentional or undetermined), the definition excludes any other external causes of death, such as road traffic and other accidents. Deaths have also been expressed as age-standardised rates. More information on how alcohol-related deaths are calculated can be found in ‘Mortality Statistics in England and Wales: Quality and Methodology Information’ [26].

#### Mean weekly alcohol consumption and exceeding the weekly recommended limits

Data on mean weekly alcohol consumption and exceeding the weekly recommended limits comes from the General Lifestyle Survey (GLS), the results of which are reported by the National Information Centre [24]. The GLS, formerly the General Household Survey, is a continuous general population survey of people living in private households in Great Britain including older adults aged 65 and over. For example, in the 2010 GLS, 18,367 individuals were interviewed of which 3,799 (21%) were aged 65 and over. The survey collects data on a range of topics including alcohol consumption.

To produce estimates of mean weekly alcohol consumption and the percentage exceeding recommended weekly amounts, respondents were asked whether they had an alcoholic drink in the last week, what type of drink they had (e.g. normal strength beer) and how much (e.g. number of large cans). In the UK, recommended weekly amounts for men are 3–4 units a day and no more than 21 units per week and for women 2–3 units a day and no more than 14 units per week. A unit of alcohol is 8 g or 10 ml of alcohol. There have been a number of methodological changes which complicate interpretation of trend data. In 2006, the average number of units assigned to the different drink types and the assumption around the average size of a wine glass was updated, resulting in significantly increased consumption estimates. Additionally, in the GLS 2008 survey, a new question was included which asked respondents about whether they had consumed small (125 ml), standard (175 ml) or large (250 ml) glasses of wine in the last week. The data from this question was used when calculating the number of units of alcohol consumed. A small glass is assumed to contain 1.5 units, a standard glass to contain 2 units and a large glass to contain 3 units. In 2006 and 2007 this calculation was different as it was assumed that all respondents drank from a standard 175 ml glass containing 2 units. In this study, data is presented based on the original methodology, as well as those based on the revised methodologies to allow meaningful comparisons across time periods.

### Secondary data analysis

#### Alcohol-related hospital admissions

Age–specific alcohol-related hospital admission rates were calculated using mid-year population estimates from the Office for National Statistics [3] for selected age groups. Age-standardised rates were standardised to the European Standard Population. Trends in alcohol-related hospital admissions for selected aged groups were determined using percentage changes between time periods.

#### Alcohol-related deaths

Age-adjusted death rates were produced for selected age groups by the Office for National Statistics. Trends were determined using percentage changes between time periods.

#### Mean weekly alcohol consumption and exceeding the weekly recommended limits

The GLS dataset was analysed using SPSS version 19. The relationship between selected age groups and mean weekly units of alcohol consumed was assessed by one-way analysis of variance (ANOVA) and the relationship between selected age groups and exceeding the recommended weekly amounts was assessed by a Chi-square test.

## Results

### Alcohol-related hospital admissions

**Figure 1:**
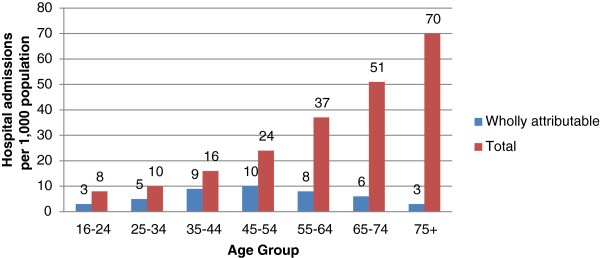
**Alcohol-related hospital admissions per 1,000 population in England, 2010/2011.**

**Figure 2:**
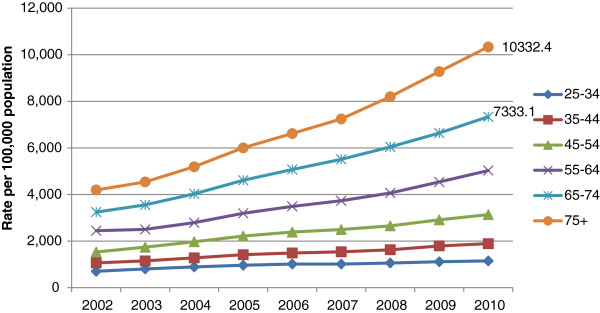
**Total alcohol-related NHS hospital admissions for men in England, 2002-2010.**

**Figure 3:**
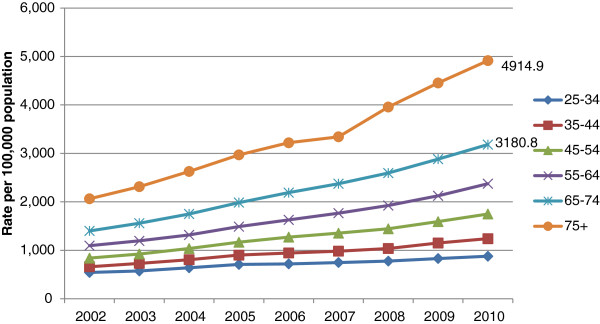
**Total alcohol-related NHS hospital admissions for women in England 2002-2010.**

**Figure 4:**
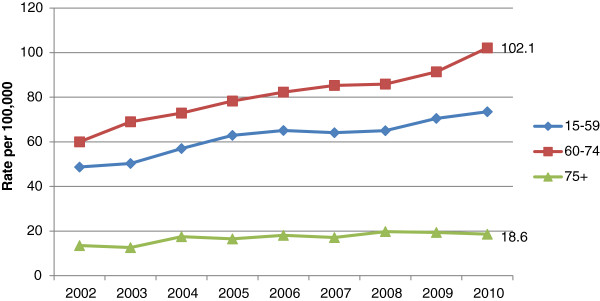
**NHS Hospital admissions for alcoholic liver disease in England 1998-2010 for selected age groups.**

Figure 1 shows wholly attributable alcohol-related hospital admissions and total alcohol-related hospital admissions (i.e. wholly and partly attributable) per 1,000 population of each age group during 2010/2011. In the 65–74 year age group, wholly attributable admissions were higher than for the 16–24, 25–34 and 75 years and over age groups but lower than in the 35–44, 45–54 and 55–64 year age groups. Wholly attributable admissions in the 75 years and over age group were the same or lower than for all other age groups. However, total alcohol-related hospital admissions increase monotonically from the youngest to the oldest age group.Figures 2 and 3 show that total age-standardised alcohol-related hospital admission rates in men and women aged 65 and over have increased markedly during the period 2002–2010. Whilst this reflects a pattern also evident in those aged 25–64, the data suggests the percentage increase in alcohol-related admissions is greater in men aged 65 and over than in men aged 25–64 (136% vs. 88%) and greater in women aged 65 and over than in women aged 25–64 (132% vs. 94%).There has also been a substantial increase in age-adjusted hospital admission rates for alcoholic liver disease amongst those aged 60–74 during the period 2002–2010 (Figure 4). This follows a similar trend to those aged 15–59 but the increase has been greatest in those aged 60–74 (70% versus 51%). Rates for the 75 and over age group have increased by 28% during this time period.

### Alcohol-related deaths

**Figure 5:**
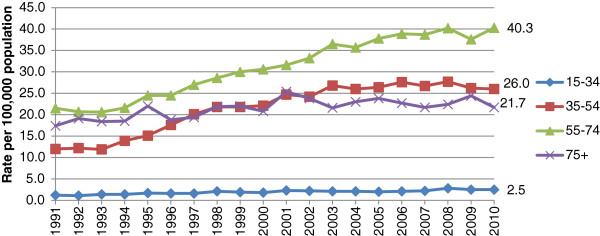
**Alcohol-related, age-standardised deaths rates for selected age groups of men, United Kingdom, 1991-2010.**

**Figure 6:**
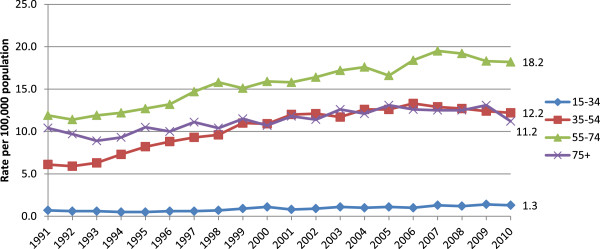
**Alcohol-related, age-standardised deaths rates for selected age groups of women, United Kingdom, 1991-2010.**

Alcohol-related, age-adjusted death rates amongst those aged 55–74 have increased by 87% for men and 53% for women during the period 1991–2010 (Figures 5 and 6) and were highest for men and women in this age category in 2010. For both men and women, alcohol-related death rates in those aged 75 and over were lower than in any age group apart from those aged 15–34 in 2010.

### Mean weekly alcohol consumption and exceeding the weekly recommended limits

**Figure 7:**
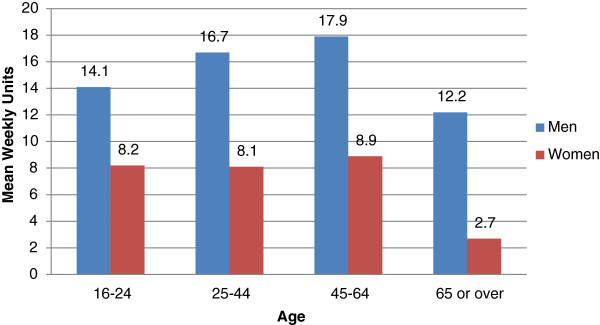
**Mean weekly units of alcohol consumed by gender and age, 2010.**

Figure 7 shows mean weekly units of alcohol consumed for selected age groups. Those aged 65 and over consumed fewer alcohol units per week on average than those aged 16–64 (8.0 units vs. 10.0 units) and one-way ANOVA showed that this difference was statistically significant (*F*(1,13236) = 127, *p* <0.0005).

**Figure 8:**
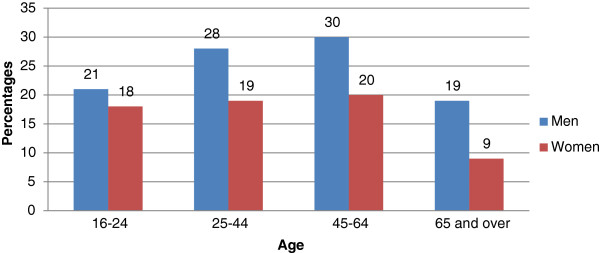
**Exceeding recommended weekly amounts by gender and age, 2010.**

Figures 8 shows the prevalence of exceeding the recommended drink limits for selected age groups. Those aged 65 and over are less likely to exceed the weekly recommended limits than those aged 16–64 (14% vs. 20%) and a chi-square test indicated that this difference was statistically significant, , χ^2^ (2, *n* = 13,238) = 181.81, *p* <0.0005.

Examining the data from previous GLS studies enables changes in alcohol consumption over time to be identified. However, the methodological changes described in the Methods section require care in interpreting trend data. Tables 1 and 2 show unweighted data for the periods 1992–1998 and weighted data for 1998 onwards. Two sets of data for 2006 and 2008 are also presented. This is to show the effect of the revised method of calculating alcohol units and the updated methodology including data on wine glass size. Using the original method of converting volumes of alcohol to units, between 1992 and 2006 mean alcohol consumption increased by 0.7 units for men aged 65 and over and 0.8 units for women aged 65 and over. Since 2008 there has been a decrease in mean weekly alcohol consumption of 1 unit for men aged 65 and over but there has been no change in the mean weekly alcohol consumption for women aged 65 and over.

**Table 1:** **Mean weekly alcohol consumption (units) for men and women aged 65 and over in Great Britain, 1992 to 2010**

	Unweighted				Weighted ^a^										
	1992	1994	1996	1998	1998	2000	2001	2002	2005	2006 original	2006 revised^b^	2008	2008 updated^c^	2009	2010
**Men**	9.7	10.0	11.0	10.7	10.6	11.0	10.8	10.7	10.4	10.4	13.5	13.6	13.2	12.8	12.2
**Women**	2.7	3.2	3.5	3.3	3.2	3.5	3.6	3.8	3.5	3.5	5.1	4.9	4.7	4.7	4.7

^a^In 2000, the decision was made to weight the data to compensate for under-representation of people in some groups. This table shows weight and unweighted data for 1998 to give an indication of the effect of weighting. Caution should be exercised when comparing weighted data with unweighted data.

^b^Figures produced using a revised methodology for converting volumes of alcohol to units assuming an average wine glass size. Two sets of data are included in the table for 2006; one is calculated using the original method and one with the improved method of calculating units.

^c^Figures produced using an updated methodology including data on wine glass size.

**Table 2:** **Weekly alcohol consumption: percentage exceeding recommended amounts for men and women aged 65 and over in Great Britain, 1992 to 2010**

	Unweighted				Weighted ^d^										
	1992	1994	1996	1998	1998	2000	2001	2002	2005	2006 original	2006 revised^e^	2008	2008 updated^f^	2009	2010
**Men**	15	17	18	16	16	17	15	15	14	14	21	22	22	20	19
**Women**	5	7	7	6	6	7	6	7	5	5	10	10	9	10	9

^d^In 2000, the decision was made to weight the data to compensate for under-representation of people in some groups. This table shows weighted and unweighted data for 1998 to give an indication of the effect of weighting. Caution should be exercised when comparing weighted data with unweighted data.

^e^Figures produced using the revised methodology for converting volumes of alcohol to units assuming an average wine glass size.

^f^Figures produced using the updated methodology including data on wine glass size.

Table 2 shows the percentage of those aged 65 and over exceeding recommended drink limits during the period 1992 to 2010. Allowing for the effect of methodological changes, there was little overall change in the percentage of men and women aged 65 and over exceeding the recommended drink limits during this period, but since 2008, the prevalence of exceeding recommended amounts for men has been decreasing.

## Discussion

Data presented here suggest that (1) older adults are more likely to be admitted to hospital for an alcohol-related condition than younger adults; (2) alcohol-related age-adjusted hospital admission rates have increased across the age-range but the most significant increases have occurred amongst older adults; (3) alcohol-related age-adjusted death rates are highest in the 55–74 year age group; (4) older adults drink less and are less likely to exceed the recommended limits than younger adults; and (5) alcohol consumption and the prevalence of excessive drinking has remained relatively stable amongst older adults in the last two decades.

It is likely that older adults are at greater risk than younger adults of being admitted to hospital for an alcohol-related condition because they have been drinking longer than younger adults and much alcohol-related harm is cumulative. Older adults’ alcohol-related disease or injuries may also be more severe and difficult-to-treat as a result of decreased physiologic reserves and co-morbidity, resulting in frequent re-admissions. It is possible that partly attributable alcohol-related hospital admissions (which make up a proportion of total alcohol-related hospital admissions) are inflated because much of the relative risk data used to determine alcohol attributable fractions is based on younger populations. Risk estimates based on older adults would allow for more precise trend comparisons to be made.

It is also possible that changes in coding practice have contributed to the apparent increase in alcohol-related admissions. For each hospital admission, clinicians record the primary diagnosis and up to 19 secondary diagnoses. An alcohol-related condition can be mentioned within the primary diagnosis field or one of the secondary diagnosis fields. There has been an increase in the coding of secondary conditions at a national level in the past decade [27]. As older adults are more likely to have more than one condition, this age group is likely to be particularly affected by the change. However, this is unlikely to be the only reason for the increase in hospital admissions in older adults because alcohol-related deaths have also increased in this age group during this period. There was a long term rise in per capita alcohol consumption since the 1960’s which peaked in 2004/2005 [28]. Changes in per capita alcohol consumption are temporally linked to changes in rates of alcohol-related harm including morbidity and mortality from various health conditions [29]. As much alcohol-related harm is the accumulated result of years of alcohol misuse, the full effect of changes in consumption are not immediately apparent in harm data. Holmes, Meier et al. [29] have demonstrated that the time to full effect is between 5 to 20 years for most alcohol-related harms. Therefore the observed increase in levels of harm in older adults is likely due to the time lag between the increase in consumption in the last 30 years and the full effect of harms being observed. Problem drinkers may also be surviving longer, resulting in more problem drinkers and alcohol-related health problems in the 65 and over age category. It is interesting that deaths rates are lowest in the 75+ age group and this may be because many chronic problem drinkers do not survive into late old age.

Data from other cross-sectional studies support the data from the GLS which suggest that older adults drink less or less problematically than younger adults [30–32]. Stall [33] has reviewed various hypotheses that might explain this observation. The mortality hypothesis proposes that heavier drinkers die earlier leaving behind lighter drinking survivors; the morbidity hypothesis suggest that older adults reduce their alcohol consumption as a result of deteriorating health; the biological hypothesis proposes that biological changes associated with ageing reduce the amount of alcohol that older adults can comfortably consume; the cohort hypothesis posits that the current cohort of older adults drinks less than later cohorts as a result of their shared experiences and the historical contexts in which they have lived rather than their life stage; the maturation hypothesis claims that alcohol problems are self-limiting; and the measurement hypothesis proposes that low prevalence rates in older adults are due to problems in accurately measuring the drinking behaviour of older adults. In support of the cohort hypothesis, cohort analysis suggests that younger birth cohorts of women in Great Britain are more likely to be heavy drinkers than members of older cohorts [34], a trend which is also evident in other countries [35]. If young women carry these relatively high levels of drinking into old age, the prevalence of problem drinking in older adults may increase.

These findings are important because they show that older adults experience a disproportionate burden of alcohol-related harm and that levels of harm are increasing more rapidly in older age groups than in younger age groups. Much of this harm could be prevented because one in three older adults with alcohol problems first develops the problem in later life [36, 37] and older adults can be successfully treated for alcohol problems. Of the 1,628 people aged 65 and over who exited an alcohol service during 2009/10, 63% were treated successfully compared to 48% of adults aged 18–64 [38]. However, older adults with alcohol problems fail to get the same attention as young people and their alcohol problems frequently remain undiagnosed. An English study reported that doctors are less likely to request an alcohol use history from older patients [39] while an Australian study found that only a third of older problem drinkers were diagnosed following hospital admission [40]. In a general hospital in the United States, over a 6 month period, medical staff correctly diagnosed only 37% of older patients with an alcohol problem compared to 60% of younger patients [41]. A survey of 597 social work and social care practitioners in England found that amongst the 85 who worked with older adults, 41% never or rarely asked their clients about alcohol or drug use and 38% said that they found it difficult to identify alcohol and drug misuse in older clients [42]. A review by the Healthcare Commission [43] found that older adults were denied access to the full range of substance misuse services because:

“Even when they were theoretically available, they were either not offered in an age-appropriate way or were not available when staff attempted to refer to them. Many were geared towards younger people, usually males, and were felt not to be appropriate for older people, who could feel vulnerable in the atmosphere”.

Social marketing programmes and alcohol strategy in the UK has primarily focused on young people and young adults.

Key priorities for action include increasing knowledge about what works in the identification, treatment and prevention of alcohol problems in older adults, increasing professionals’ competencies and skills in identifying and working with older adults with alcohol problems, ensuring that alcohol treatment services are sensitive to the needs of older adults and ensuring that national campaigns and strategy is inclusive of older adults. Given the current population projections, taking action on alcohol misuse in older adults can be regarded as a sound investment that will have long term value for subsequent generations.

## Conclusion

Older adults experience high levels of alcohol-related harm but drink less than younger adults and levels of alcohol-related harm in older adults are increasing. Careful monitoring of alcohol use and related harm amongst older adults is required to see if current trends will continue. This information will aid decision making about priorities for action and resource allocation.
